# Antibiogram profile and virulence signatures of *Pseudomonas aeruginosa* isolates recovered from selected agrestic hospital effluents

**DOI:** 10.1038/s41598-021-91280-6

**Published:** 2021-06-03

**Authors:** Q. Mapipa, T. O. Digban, N. E. Nnolim, U. U. Nwodo

**Affiliations:** 1 SA-MRC Microbial Water Quality Monitoring Centre, University of Fort Hare, Alice, 5700 South Africa; 2 Applied and Environmental Microbiology Research Group (AEMREG), Department of Biochemistry and Microbiology, University of Fort Hare, Private Bag X1314, Alice, 5700 Eastern Cape South Africa

**Keywords:** Microbiology, Molecular biology

## Abstract

Hospital wastewater (HWW) harbours diverse microbial species and a miscellany of genome that would facilitate the emergence of novel pathogen upon genome integration that manifests novel traits in infectious pathogens. The study aimed to determine the antibiogram, and virulence signatures of *Pseudomonas aeruginosa* (*P. aeruginosa*) recovered from selected agrestic hospital effluents in Eastern Cape, South Africa. Thirty-six (36) wastewater samples were collected from selected hospital drains between February 2018 and April 2018, processed and analyzed by culture-dependent methods for the isolation of *P. aeruginosa*. The identity confirmation of isolates was achieved by amplification of *oprl* and *oprL* genes. Antibiogram was done using standard disk diffusion technique of Kirby–Bauer as approved by CLSI 2018 guidelines. Virulence signatures (*lasA, lasB, toxA, popB*) among isolates were analysed using polymerase chain reaction. A total of 54 *P. aeruginosa* isolates were confirmed by amplification of *oprl* and *oprL* genes in the hospital wastewater effluent samples. The isolates showed a 100% susceptibility to gentamicin, amikacin and imipenem antimicrobial agents. Ceftazidime recorded the most resistance (63%) against the isolates studied. Other antibiotics had a resistance range of 7% and 35%. The MAR index among the isolates revealed a range of 0.23 and 0.38. *ToxA* virulence gene was detected in all isolates while *popB, lasB, lasA* were detected in 82%, 75% and 54% of the isolates. This study reveals *P. aeruginosa* isolates with virulence traits and some strains showing multiple antibiotic resistance. The multiple antibiotic resistance index (MARI) of ≥ 0.2 indicates that the some isolates may have emerged from high-risk sources, thus projecting a risk to public health. However, with the high sensitivity pattern observed among the studied isolates, most of the antibiotics used in the susceptibility tests are not at peril. Hence, the use of these antibiotics is encouraged for treatment of infection attributed to *P. aeruginosa*. It is also pertinent to initiate strict control and rigid antibiotics therapeutic policy with surveillance programmes for multidrug-resistant pathogens to forestall the development and transmission of resistance traits in the pathogens.

## Introduction

Hospital wastewaters (HWW) are among the probable puddle through which antimicrobial-resistant bacterial strains emerge^1^. Hospitals are epidemiologically significant focal points for all kinds of pathogens. HWW has been akin to the cesspool of the microbial genome where interaction and recombination occur, leading to the emergence of pathogens with modified or novel traits. Antibiotic consumption and resistance are increasing considerably due to the rapid economic and growing population as well as increasing burden of infectious ailments^2^. Antibiotics in wastewater originate from discarded expired drug, accidental spilling of medications and excretion of drugs in urine or faeces, all of which ostensibly serve as additional selective pressure on bacteria while in wastewater. Hospital effluents have been recognized to be enriched with antibiotic-resistant pathogens, including opportunistic *P. aeruginosa*^3,4^*. P. aeruginosa* is a Gram-negative, aerobic rod bacterium of the *Pseudomonadaceae* family. The outer membrane proteins *oprI* and *oprL* codes for membrane integrity and normal cell shape significantly used as taxonomic tools for *P. aeruginosa* identification and delineation^5^. *P*. *aeruginosa* genome size has been approximated as 5 to 7 million base pair (Mbp) and substantial proportions of their conserved genes encode regulatory proteins. The implication is that *P*. *aeruginosa* can adjust to several environmental stressors^6^. *P. aeruginosa* is an opportunistic pathogen, ubiquitous and typically resides in the soil, surfaces and aqueous environments. They are not human commensal and can infect virtually most tissues of their host^7^. Infections attributed to *P. aeruginosa* are seldomly life-threatening. However, intrinsically high resistance to numerous antimicrobial agents resulted in the emergence of increased multidrug resistance strains. Bacteria are known to exhibit multiple resistance mechanisms to antibiotics with decreased outer membrane permeability, exhibition of efflux systems that expels dugs out of the cells, production of antibiotic inactivating enzymes and target modifications^8,9^. *P. aeruginosa* displays most of these known resistance mechanisms through intrinsic chromosomally encoded or genetically acquired resistance traits impeding the major classes of antibiotics such as β-lactams, aminoglycosides, quinolones and polymyxins^8^. *P. aeruginosa* harbours an arsenal of virulence genes that assist in facilitating infection and colonisation across a wide range of environments. Virulence factors such as type III secretory proteins (*exoT*, *exoS*, *exoY*, and *exoU*), regulate the expression of exotoxins, quorum-sensing (QS) system proteins (*lasR/lasI* and *rhlR/rhlI*) permits the interaction of cells. Others include elastases (*lasA* and *lasB*) that distorts bonded links between host epithelial cells, alginate (*alg* genes), and pigments such as pyoverdine regulating the transcription associated with oxidative stress stimulating the modification of mitochondrial electron transport of the host^10^. *P. aeruginosa* infections are frequently hospital-acquired and most are associated with immunocompromised individuals. Environmental contamination and direct transmission from patients or personals in healthcare are often the reservoirs of *P. aeruginosa* in health institution settings^11^. Some *P. aeruginosa* virulence factors are tightly regulated by cell-to-cell signalling systems, and others are involved in toxicity by induction of apoptosis within the host cell^12^. Several reports have identified HWW as an ideal puddle for microbial interaction with consequential public health significance^13–15^. Therefore, on the strength of the advanced concept, it was imperative to evaluate the antibiogram, and virulence signatures of *P. aeruginosa* isolates recovered from HWW effluents in selected agrestic hospitals in the Eastern Cape Province of South Africa.

## Results

**Figure 1:**
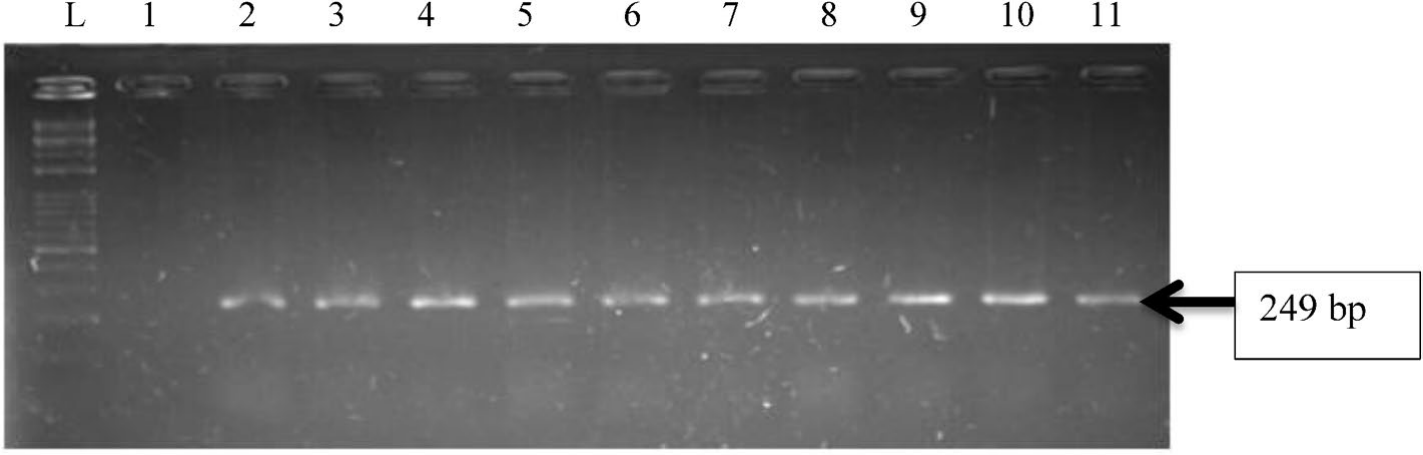
Gel electrophoresis of PCR products of *oprl* gene among *P. aeruginosa* representatives isolates recovered from HWW effluent. Lane 1: DNA Marker (100 bp). Lane 1: negative control. Lane 2–11: positive isolates of *oprl* gene (249 bp).

**Figure 2:**
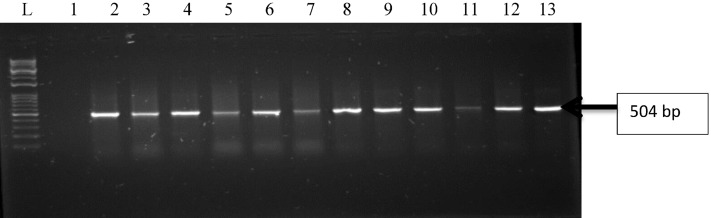
Gel electrophoresis of PCR products of *oprL* gene among *P. aeruginosa* representatives isolates recovered from HWW effluent. Lane 1: DNA Marker (100 bp). Lane 1: negative control. Lane 2–13: positive isolates of *oprL* gene (504 bp).

HWW effluent samples harboured *P. aeruginosa*. Over the twelve weeks (three months) sampling period, approximately 174 presumptive *P. aeruginosa* was recovered from the HWW effluents, and 54 were confirmed molecularly to comprise 20% from hospital A, and 80% from hospital B. Isolates belonging to *P. aeruginosa* were not recovered from hospital C. The total number of affirmed *P. aeruginosa* isolates from the three hospitals is shown in Table 1. Confirmation of isolates identity through amplification of *oprl* and *oprL* genes is represented in Figs. 1 and 2.

**Table 1:** Total number of *P. aeruginosa* isolates recovered from the three hospital effluents.

Organism	Presumptive isolates	Confirmed isolates	Hospital A %	Hospital B %	Hospital C %
*Pseudomonas aeruginosa*	174	54	11(20)	43(80)	0(0)

The 54 confirmed isolates were tested against six classes of antimicrobial agents. Within the aminoglycoside class, all isolates exhibited a 100% sensitivity rate to both gentamicin and amikacin, while 93% sensitivity rate was observed with tobramycin antibiotic. Among the fluoroquinolones antimicrobial class, a high sensitivity rate was observed for ciprofloxacin (91%), norfloxacin (89%) and ofloxacin (91%). The carbapenems also exhibited high sensitivity rates with all isolates presenting 100% sensitivity to imipenem, while 93% and 96% sensitivity rate was observed in doripenem and meropenem antibiotics, respectively. However, 63% resistance to ceftazidime and (35%) resistance to cefepime were observed among the isolates. Apparently 87% and 81% of the isolates showed sensitivity to penicillins and beta lactam/lactamase inhibitors. Moreover, ceftazidime and cefepime recorded the highest levels of resistance observed in this study. The distribution of Antimicrobial susceptibility of *P. aeruginosa* isolates is displayed in Table 2.

**Table 2:** Distribution of the Antimicrobial Susceptibility Pattern of *P. aeruginosa* isolates.

Antimicrobial class	Antibiotics	Isolates (n = 54)		
		S	I	R
Aminoglycosides	Gentamicin (10 μg)	54(100)	0(0)	0(0)
	Tobramycin (10 μg)	50(93 )	0(0)	4(7)
	Amikacin 10 μg)	54(100)	0(0)	0(0)
Fluoroquinolones	Ciprofloxacin (5 μg)	49(91)	1(2)	4(7)
	Norfloxacin (10 μg)	48(89)	2(4)	4(7)
	Ofloxacin (5 μg)	49(91)	0(0 )	5(9)
Carbapenems	Imipenem (30 μg)	54(100)	0(0)	0(0)
	Doripenem (30 μg)	50(93)	0(0)	4(7)
	Meropenem (30 μg)	52(96)	0(0)	2(3)
Cephalosporins	Ceftazidime (30 μg)	20(37)	0(0)	34(63)
	Cefepime (30 μg)	35(65)	0(0)	19(35)
Penicillins	Piperacillin (100 μg)	47(87)	0(0)	6(11)
β- lactamase inhibitor	Piperacillin-tazobactam (100/10 μg)	44(81)	2(3)	8(15)

*R:* Resistant, *S:* Sensitive, *I:* Intermediate, *n:* Number of isolates, the parenthesis value denotes percentage (%).

Multiple antibiotics resistance index was determined with the formula: “MARI = a/b.

Where (a) is the number of antibiotics which the isolates showed resistance, (b) is the total number of antibiotics used in each class of antimicrobial agent”^16^. MARI of ≥ 0.2 infers that the strain of such bacteria originate from an environment where several antibiotics are being used. The MAR index from our study revealed slight variations with the lowest MAR index of 0.23 and the highest MAR index of 0.38. However, more than half of the isolates had MAR1 of 0.31 as shown in Table 3. Isolates that showed resistance to two or more class of antibiotics were regarded as showing multi-drug resistance.

**Table 3:** Multidrug resistance profile of the *P. aeruginosa* isolates.

Phenotypic resistance	Number of isolates	MAR index
TOB^R^ , CIP^R^, NOR^R^	3	0.23
TOB^R^ , CAZ^R^, FEP^R^	12	0.23
CIP^R^, NOR^R^, DOR^R^, FEP^R^	7	0.31
TOB^R^, DOR^R^, CAZ^R^, PIP^R^	11	0.31
CIP^R^, DOR^R^, FEP^R^, TZP^R^	7	0.31
NOR^R^, DOR^R^, FEP^R^, PIP^R^	7	0.31
TOB^R^, CIP^R^, MEM^R^, CAZ^R^, TZP^R^	11	0.38
MEM^R^, CAZ^R^, PIP^R^	9	0.23
OFX^R^, CAZ^R^, PIP^R^	13	0.23
NOR^R^, FEP^R^, TZP^R^	10	0.23

*TOB* tobramycin; *CIP* ciprofloxacin; *NOR* norfloxacin; *CAZ* ceftazidime; *FEP* cefepime; *DOR* doripenem; *PIP* piperacillin; *TZP* piperacillin-tazobactam; *MEM* meropenem; *OFX* ofloxacin; *IPM*: imipenem; *AMK* amikacin; *GEN* gentamicin.

**Figure 3:**
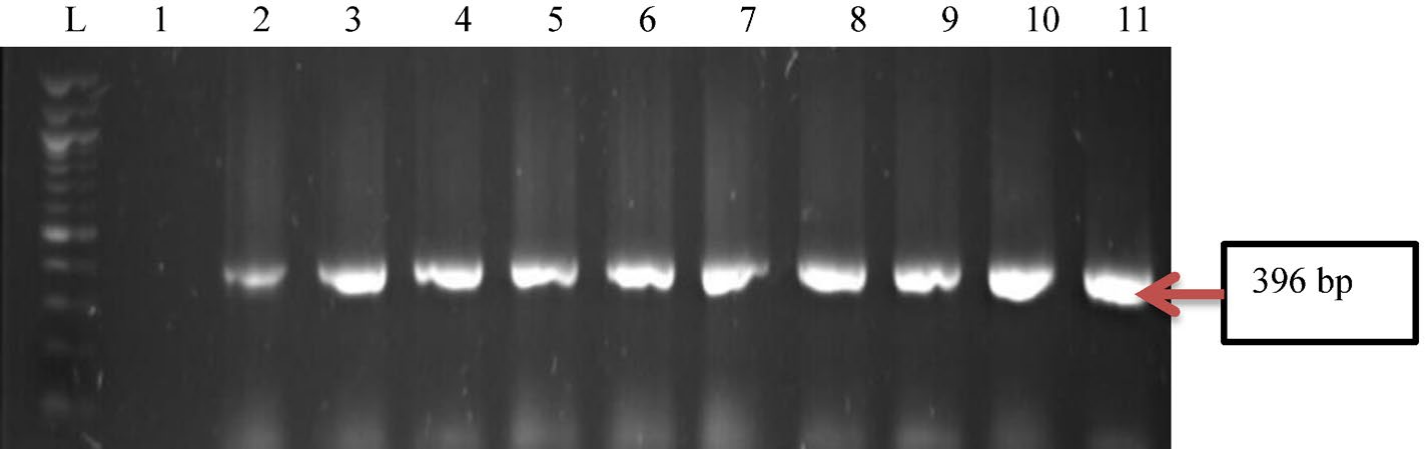
Gel electrophoresis of PCR products of *toxA* gene among *P. aeruginosa* representatives isolates recovered from HWW effluent. Lane 1: DNA Marker (100 bp). Lane 1: negative control. Lane 2–11: positive isolates of *toxA* gene (396 bp).

**Figure 4:**
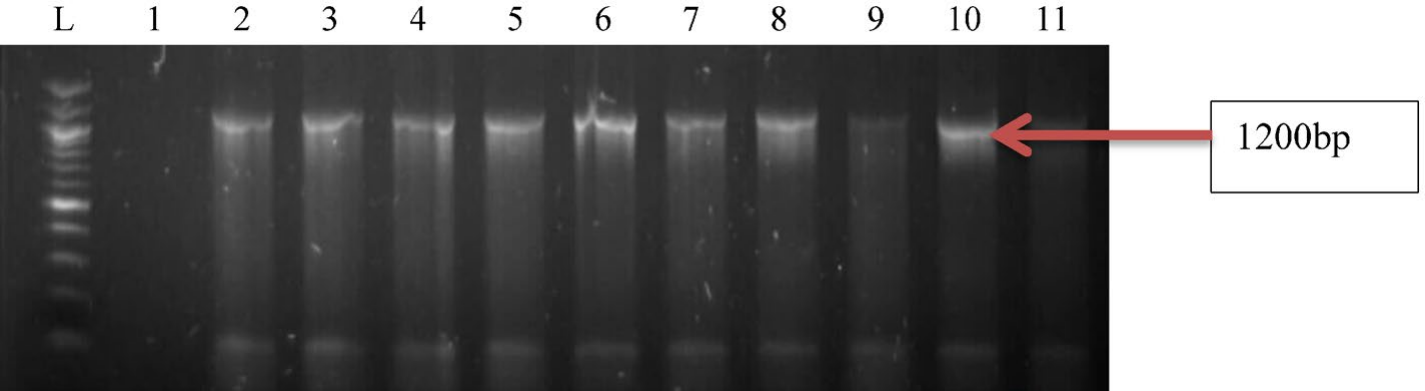
Gel electrophoresis of PCR products of *popB* gene among *P. aeruginosa* representatives isolates recovered from HWW effluent. Lane 1: DNA Marker (100 bp). Lane 2–11: positive isolates of *popB* gene (1200 bp).

**Figure 5:**
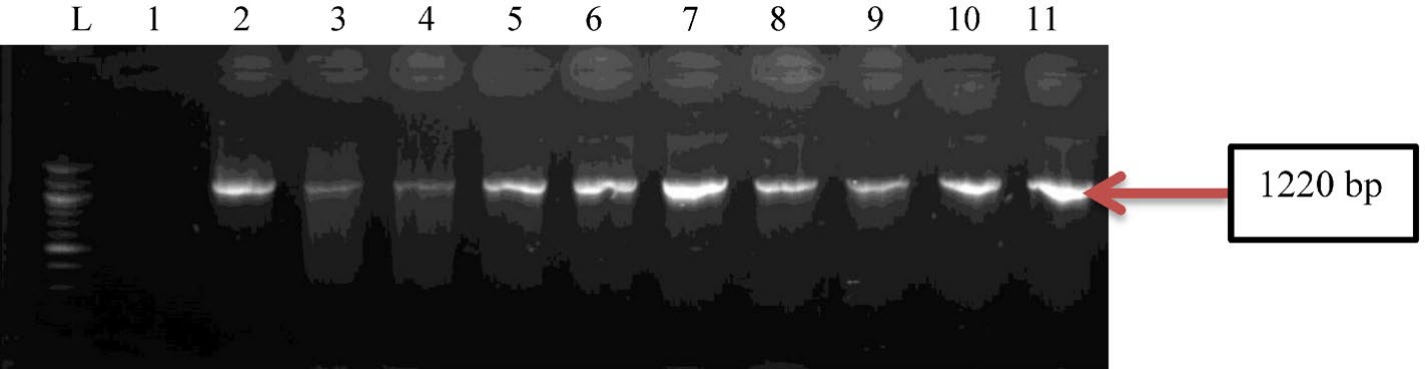
Gel electrophoresis of PCR products of *lasB* gene among *P. aeruginosa* representatives isolates recovered from HWW effluent. Lane 1: DNA marker (100 bp). Lane 2–11: positive isolates of *lasB* gene (1220 bp).

**Figure 6:**
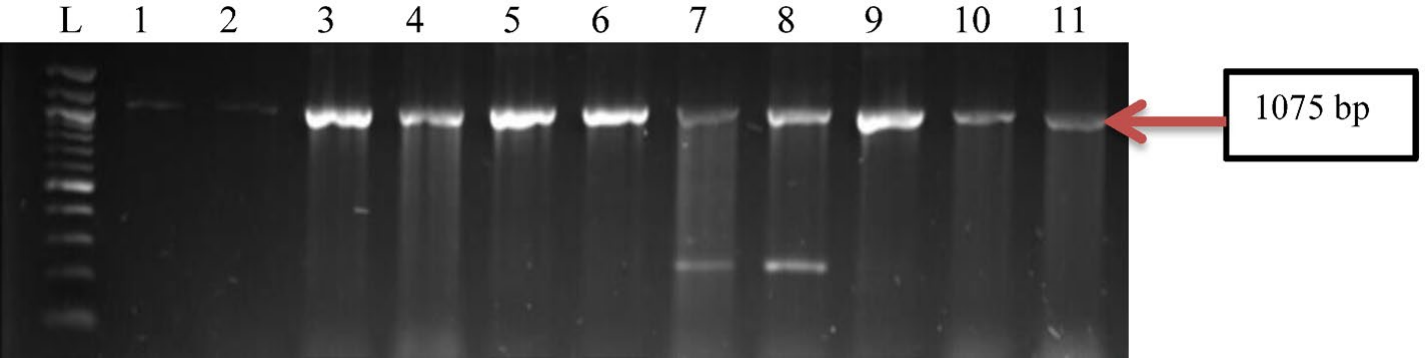
Gel electrophoresis of PCR products of *lasA* gene among *P. aeruginosa* representatives isolates recovered from HWW effluent. Lane 1: DNA marker (100 bp). Lane 1–11: positive isolates of *lasA* gene (1075 bp).

**Figure 7:**
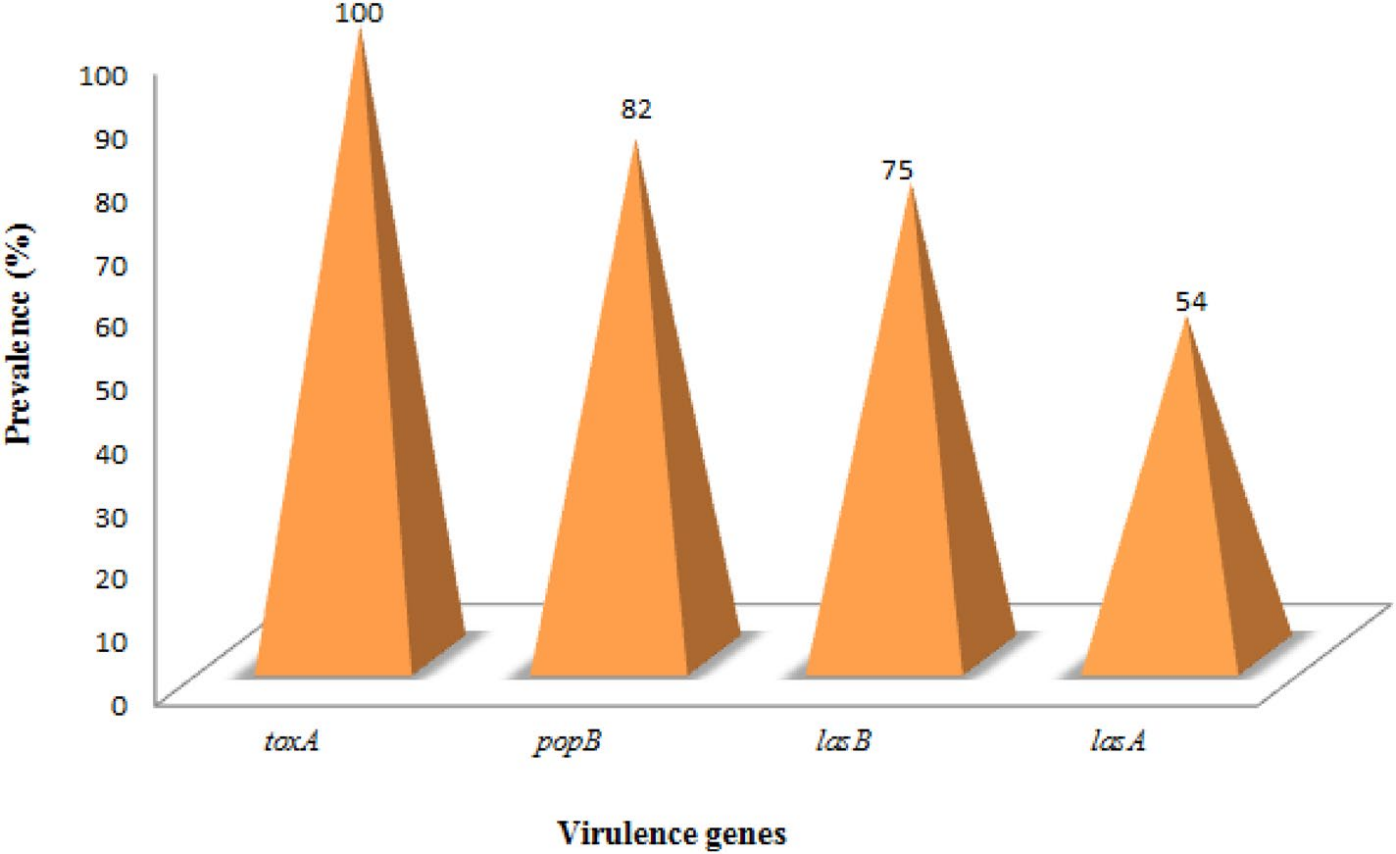
Prevalence of virulence genes among all confirmed *P. aeruginosa* isolates.

All confirmed positive isolates of *P. aeruginosa* were screened for the presence of some virulence determinants using PCR methods. Findings from this study revealed that the isolates harboured all four of the virulence genes in the following proportions, *toxA* (100%), *popB* (82%), *lasB* (75%), and *lasA* (54%), been *toxA* the most virulence gene detected (Figs. 3, 4, 5, 6, 7).

The frequency of the virulence genes among the isolates was also enumerated. 39% of the isolates harboured all four genes, 31% of the isolates harboured three genes and 24% harboured only two genes while only an isolate (QKP32) harboured a single gene. The distribution of virulence genes among the *P. aeruginosa* isolates is shown in Table 4.

**Table 4:** Distribution of virulence genes among the *P. aeruginosa* isolates.

Isolate code	*toxA*	*popB*	*lasB*	*lasA*
QKP1	+	+	+	+
QKP2	+	+	−	−
QKP3	+	+	+	+
QKP4	+	+	+	−
QKP5	+	+	+	+
QKP6	+	−	+	+
QKP7	+	−	+	−
QKP8	+	−	+	−
QKP9	+	+	+	+
QKP10	+	+	−	−
QKP11	+	+	+	+
QKP12	+	−	−	+
QKP13	+	+	−	+
QKP14	+	+	+	+
QKP15	+	−	−	+
QKP16	+	+	+	+
QKP17	+	+	+	+
QKP18	+	+	+	+
QKP19	+	+	+	+
QKP20	+	+	+	+
QKP21	+	+	+	+
QKP22	+	+	+	−
QKP23	+	+	+	−
QKP24	+	+	−	+
QKP25	+	+	+	+
QKP26	+	−	+	−
QKP27	+	+	+	−
QKP28	+	+	+	−
QKP29	+	+	−	−
QKP30	+	+	+	+
QKP31	+	+	+	+
QKP32	+	−	−	−
QKP33	+	+	+	−
QKP34	+	−	+	−
QKP35	+	+	+	+
QKP36	+	+	+	−
QKP37	+	+	−	−
QKP38	+	+	+	−
QKP39	+	+	+	−
QKP40	+	+	−	−
QKP41	+	+	−	−
QKP42	+	+	+	−
QKP43	+	+	+	−
QKP44	+	+	+	−
QKP45	+	+	+	+
QKP46	+	+	+	+
QKP47	+	+	+	+
QKP48	+	+	−	+
QKP49	+	−	+	+
QKP50	+	+	+	+
QKP51	+	−	−	+
QKP52	+	+	+	+
QKP53	+	+	+	+
QKP54	+	+	+	−

## Discussion

Hospitals have a significant role in the well-being of humankind, including other health research advancement. Various units/services in hospitals require copious water volume relative to the many activities within the hospitals and thus generate a large amount of wastewater^17^. The characteristic of hospital wastewater is quite different from the wastewater discharged from other sources in harbouring a wide range of infectious microbe. The effluent from hospitals is directly discharged into the municipal sewer system co-treated with urban wastewater. HWW poses a grave risk to humans as it radically disseminates infectious pathogens found in healthcare wards to the environment^18^. The dissemination of antibiotic non-susceptible bacteria, their reservoirs and distribution in the environment are very pertinent issues. There are numerous reports of *P. aeruginosa* disease outbreak that has been attributed to both hospital-acquired and environmental sources associated with serious infections in the immune-compromised host, severe burns patients and those with surgical injuries^19–21^. *P. aeruginosa* is pervasive, and can survive in the environment over a long time owing to its proclivity to utilize a vast array of organic material as energy sources^22^. Apparently, accurate and rapid identification of *P. aeruginosa* and knowledge of the susceptibility profile of this organism is significant. This may be useful in avoiding prolonged and occasionally unnecessary antibiotic treatments, which could be selected for other antibiotic resistant pathogens. Molecular methods have been documented to be superior to the phenotypic methods in identifying and characterization of *P. aeruginosa* strains. The phenotypic based approaches are part of the traditional typing methods such as biochemical profiling, serotyping, phage typing, and pyocin typing^23^. However, their biased is much lower by molecular methods like restriction fragment length polymorphic DNA analysis, pulse-field gel electrophoresis, polymerase chain reaction, real time polymerase chain reaction as well as next generation sequencing^24,25^. PCR assay was performed independently in this study for the molecular detection of two outer membrane lipoprotein genes of *P. aeruginosa* known as *oprI* and *oprL*. All of the isolates were remarkably positive for both *oprI* and *oprL* genes. The outer membrane proteins of *P. aeruginosa* play an important role in the interaction of the bacterium with the environment. *Oprl* gene has previously been identified as a conserved region in members of all fluorescent *Pseudomonas* while *oprL* had been used for the detection of species of *P. aeruginosa* strains^26,27^. *P. aeruginosa* holds an array of arsenal with extracellular virulence traits that include lipases, proteases, pyocyanin and secretion toxins which are known for initiating pathogenicity^28^. This study is in agreement with a previous study that revealed low recovery of *P. aeruginosa* from hospital effluents^29^.

This study also displays a high level of sensitivity pattern to the five classes of antimicrobial agents employed. The aminoglycosides, carbapenems and fluoroquinolones exhibited a very high sensitivity pattern to the isolates. Antibiotics belonging to other antimicrobial classes from our study showed less resistance to the isolates except CAZ and FEP which are third and fourth-generation cephalosporins, respectively. Both antibiotics showed the highest levels of resistance in this study. Isolates possessing the beta-lactamase enzymes are able to inactivate these drugs, hence resulting in their high resistance. In this context, the primary source of cephalosporin resistance in *P. aeruginosa* isolates is the excessive expression of the chromosomal AmpC β-lactamases. Moreover, Mutation-dependent overproduction of intrinsic β-lactamase AmpC has been identified as one of the main causes of resistance of clinical strains of *P. aeruginosa*^30^. Furthermore, other β-lactamases have been identified in *P. aeruginosa*, as an effect of horizontal gene transfer, having different substrates and inhibitor profiles^9^. Some *P. aeruginosa* isolates have been reported to produce ESBLs which are mostly in enzyme class A beta-lactamases, conferring high degree of resistance to cephalosporins. Isolates possessing the beta-lactamase enzymes are able to inactivate these drugs, hence resulting in their high resistance. The isolates from our study also showed high sensitivity pattern to fluoroquinolones, penicillins, beta-lactamase inhibitors and similar to other previous studies^31,32^. Our study is in contrast with another report^33^ which had almost all the strains exhibiting varying degree of resistance to the antibiotics tested. Despite the low resistance profile observed by the isolates in the study, some strains also exhibited multiple drug resistance in agreement with a finding^34^ which had over 40% of the isolates exhibiting multi‐drug resistance The multiple antibiotic resistance end point index among the isolates had a range of 0.23 and 0.38. These values indicate that the isolates may have developed resistance through inherent chromosomal mechanism or through plasmid mediated route. Horizontal transfer with other competent microbes, selective drug pressures and persistent drug use are among other factors that would probably have induced the resistance.

Production of virulence traits is a survival approach for pathogens to evade the host immune mechanism resulting in pathogenesis mostly at the initial stage of colonization and acute infection. A great amount of virulence factors as well as cell-associated or secreted compounds of minimal and high molecular weight have been documented as vital in establishing infections by *P. aeruginosa*^35,36^. Though they perform important roles in encouraging bacterial growth and persistence, they can induce fatal damages to the host tissues and weaken the immune reactions^37^. Some virulence genes were screened among the isolates based on previous studies on detection of virulence genes among *P. aeruginosa* from clinical samples. *ToxA* is an inherent genetic fragment located on the chromosome of *P. aeruginosa* is known for regulating the synthesis of exotoxin A. However, exotoxin A is a significant virulence factor with its role in clinical infections having cytotoxic effect that inhibits the biosynthesis of protein at the phase of elongation factor 2 in the polypeptide chain resulting in huge organ and tissue loss^38^. All recovered isolates from hospital effluent in this study harboured the *toxA* gene and concurs with previous study^39^. *LasB* also known as elastase B mediates the invasiveness of *P. aeruginosa* and shown to be highly toxic to the host through its enzymatic activity to impair numerous mechanisms of innate and adaptive immune systems^40–42^. They also cause host tissue damage via hydrolysis of various components of the extracellular matrix and by breaching endothelial and epithelial barriers leading to the attack of intercellular tight junctions^43^. *LasB* gene was detected in 75% of the isolates studied and is in accord with several previous studies^44–47^. *PopB* gene codes a protein needed for appropriate translocation of effector proteins^48^. From our study, 82% of the isolates harboured the *popB* gene and higher than the precious findings^49^. *LasA* (elastase A) belongs to the beta-lytic family of ZINC-metallo-endopeptidases, with high staphylolytic activity and also responsible for shedding of the host cell surface proteoglycan syndecan-1^50^. Our data also showed that *lasA* gene was detected in 54% of the isolates. The production of elastase protein is regulated by several factors including the growth rate of *P. aeruginosa.* Result from this study is in agreement with previous studies^51,52^ which detected *lasA* gene in over 70% of *P. aeruginosa* isolates. Pathogenicity of *P*. *aeruginosa* is multifactorial; the detection of different virulence genes in *P. aeruginosa* isolates suggests that they can be linked with different levels of inherent virulence and their propensity to cause infection. The correlation of multi-drug resistance and virulence gene was not ascertained in the study. However it is worthy to note that virulence and other phenotypic traits like resistance genes can contribute to the survival of organism, as well as in disease spread and severity.

Studies comprising analysis of effluents from more hospitals are highly recommended in order to better establish *P*. *aeruginosa* transmission in many geographical region, as well as, showing the associations between of virulence, antibiotic susceptibility and genetic diversity among the isolates.

## Conclusion

This study reveals low recovery of *P. aeruginosa* from the hospital wastewater sampled. However, these isolates also harboured virulence traits (*popB, lasB, lasA* and *toxA)* that may encourage their adaptability in the environment and exert pathogenicity to susceptible host. Some strains from the study also presented multiple antibiotic resistances which could pose public health risk and also pave way for the influx of new antimicrobial agents to thwart the emergence of resistance strains. Nonetheless, with the high susceptibility pattern (gentamicin, amikacin and imipenem) observed among the isolates to the antibiotics, continuous use of most of the antibiotics in this study (with the exception of ceftazidime) should be encouraged for patients in the hospital as well as observing their non-abuse. It is pertinent that antimicrobial susceptibility test on pathogens like *P. aeruginosa* be continuously monitored to determine antibiotic resistance profile as a useful index to control the emergence of isolates in hospital effluents. It is also imperative to have strict control and rigid antibiotics therapeutic policy with surveillance programme for multidrug resistance pathogens to prevent transmission of resistance genes to other pathogenic or commensals in the hospital wastewater effluents.

## Materials and methods

The sampling was carried out between February 2018 and April 2018 in some selected agrestic hospital within the Eastern Cape. Strict confidentiality was observed, and the three hospitals for which wastewater effluents samples were collected were designated as A, B and C. The hospitals are located in Amatole District Municipality, bordering the Nxuba Municipality to the west and the Amahlathi Municipality to the east. The municipality has a largely rural population with more than 20 wards, and the hospitals were selected based on the patients’ outflow and their medical facilities.

### Collection of sample

Thirty-six (36) wastewater samples from hospital drains were obtained in three different healthcare drains. Twelve (12) wastewater samples were obtained from each designated hospitals between February 2018 and April 2018. Wastewater effluents from the hospital drains were carefully collected into sterile 1L propylene glass bottles. The samples were appropriately labelled and placed in coolers containing ice packs for onward transportation to the Applied and Environmental Microbiology Research Group (AEMREG) laboratory at the University of Fort Hare for immediate analysis.

### Sample processing and cultivation

The wastewater samples were processed according to Standard Methods^53^. Each sample was serially diluted tenfold by adding 20 ml of the wastewater samples to 180 ml of sterile distilled water. Following dilutions, samples were filtered through 0.45 μm mixed cellulose ester membrane filters (Whatman, Piscataway, NJ) using a vacuum pump. Filters were carefully impregnated on prepared Centrimide agar plates and incubated at 37 °C for 24–48 h. After incubation, colonies showing green colour with raised mucoid appearance were considered as presumptive *Pseudomonas* positive isolates.

### DNA extraction

Chromosomal nucleic acid from the presumptive isolates was extracted by the boiling method as described previously^54^ but with slight modification. Briefly, few colonies of bacterial strain were suspended in 250 μl of phosphate buffered saline (PBS) in microcentrifuge tubes and spun at 13,000 rpm to wash the cells. The supernatant was decanted gently leaving the pellets to settle at the bottom of the tube and 200 μl of sterile distilled water added. This was mixed for few seconds and placed in a heating block set at 100 °C for 10 min followed by centrifugation at 13,500 rpm for another 10 min, and the supernatant was carefully pipetted into a sterile Eppendorf before storage at − 20 °C until use.

### Molecular identification of Pseudomonas aeruginosa

The molecular PCR was done in a total reaction mixture of 25 μl comprising 12.5 μl of commercially synthesized master mix (thermo scientific, USA), 1ul each of forward and reverse working stock primers (integrated DNA Technologies, USA), 6.5 μl of PCR grade water and 4 μl of the template DNA. Primer name, oligonucleotide sequences and product size are shown in Table 5.

**Table 5:** Oligonucleotide primers used for the amplification of *oprl* and *oprL* genes.

Primer	Oligonucleotide primer (5′ → 3′)	Target gene	Fragment size (bp)	Ref
PS1-F PS2-R	ATGAACAACGTTCTGAAATTC CTTGCGGCTGGCTTTTTCCA	*Oprl*	249	^55^
PAL1-F PAL1-R	ATGGAAATGCTGAAATTCGGC CTTCTTCAGCTCGACGCGACG	*OprL*	504	^55^

The PCR was set up in a thermocycler (Bio-Rad, T100 thermal cycler Singapore) with cycling conditions for *oprl* and *oprL* genes as described previously but with slight adjustment as follows: Initial denaturation of 94 °C for 5 min, and 35 cycles consisting of 94 °C denaturation for 40 s, annealing for 40 s at 57 °C, elongation at 72 °C for 1 min and a final extension for 10 min at at 72 °C. Amplicons were checked for band size using gel electrophoresis with 1.5% (w/v) agarose gel and 0.5 × Tris- EDTA buffer, run at 100 V for 55 min. The gel was stained with 4 μL ethidium bromide (0.5 μg/ml) and 100 bp DNA marker (Fermentas, Lithuania) was used as the DNA ladder and then viewed under UV transilluminator (Alliance 4.7, UVITEC, Cambridge).

### Susceptibility test

The antibiotic susceptibility was tested on Mueller–Hinton agar (Basingshike, Hampshire, England) plates by the standard disk diffusion technique (Kirby-Bauer test) as recommended by the Clinical and Laboratory Standards Institute^56^ (CLSI 2018). Briefly, the confirmed *P. aeruginosa* were grown on Mueller-Hilton agar and incubated at 37 °C for 18–24 h. After incubation, few colonies were reconstituted on sterile physiological saline to a diluent approximating to 0.5 McFarland standards. The bacterial suspension was spread onto a Mueller–Hinton agar plate surface to form a confluent lawn and incubated for approximately 15 min, afterwards, the agar plates were impregnated with the following antibiotic discs; Amikacin (30 μg), Gentamicin (10 μg), Tobramycin (10 μg), Ceftazidime (30 μg), Cefepime (30 μg), Doripenem (30 μg), Imipenem (30 μg), Meropenem (30 μg), Piperacillin (100 μg), Ciprofloxacin (5 μg), Levofloxacin (5 μg), Ofloxacin (5 μg), and Piperacillin-tazobactam (100/10 μg). Plates were read after 24 h incubation and results recorded.

### Virulence genes identification

The evaluation for the presence of virulence gene in *P. aeruginosa* was done using conventional polymerase chain reaction technique with specific synthesized primers as shown in Table 6. The targeted virulence genes include *ToxA, LasA, LasB,* and *popB* and the molecular cycling condition for the virulent genes are as stated; a 5 min initial denaturing at 94 °C followed by simplex PCR conditions, 35 cycles of 94 °C for 1 min, annealing temperature (51 °C, 55 °C, 57 °C and 68 °C) based on the different virulence genes was set at 1 min and elongation at 72 °C for 1 min. This was followed by a final extension of 72 °C for 10 min. The final amplified products were electrophoresed through agarose gels (1%) containing 0.5% ethidium bromide and visualized under UV transilluminator.

**Table 6:** Oligonucleotide primers used in the detection of virulence genes for *P. aeruginosa.*

Primer Sequences (5′ → 3′)	Target gene	Fragment size (bp)	Annealing temp °C	Ref
F-GACAACGCCCTCAGCATCACCAGC R-CGCTGGCCCATTCGCTCCAGCGCT	*Tox A*	396	68	^57^
F-GCAGCACAAAAGATCCC R-GAAATGCAGGTGCGGTC	*LasA*	1075	57	^57^
F- ACAGGTAGAACGCACGGTTG R- GATCGACGTGTCCAAACTCC	*LasB*	1220	50	^58^
F- TTTGGATCCATGAATCCGATAACGCTT R- TTTGAATTCTCAGATCGCTGCCGGTCG	*PopB*	1200	55	^48^

## Data Availability

The data and materials used during the current study are available upon request to the corresponding author.
